# Signal Regulatory Protein α (SIRPα)^+^ Cells in the Adaptive Response to ESAT-6/CFP-10 Protein of Tuberculous Mycobacteria

**DOI:** 10.1371/journal.pone.0006414

**Published:** 2009-07-29

**Authors:** W. Ray Waters, Mitchell V. Palmer, Brian J. Nonnecke, Tyler C. Thacker, D. Mark Estes, Michelle H. Larsen, William R. Jacobs, Peter Andersen, James McNair, F. C. Minion, Konstantin P. Lyashchenko, R. Glyn Hewinson, H. Martin Vordermeier, Randy E. Sacco

**Affiliations:** 1 National Animal Disease Center, Agricultural Research Service, U.S. Department of Agriculture, Ames, Iowa, United States of America; 2 University of Texas Medical Branch, Department of Pediatrics and the Sealy Center for Vaccine Development, Galveston, Texas, United States of America; 3 Howard Hughes Medical Institute and Department of Microbiology and Immunology, Albert Einstein College of Medicine, Bronx, New York, United States of America; 4 Statens Serum Institute, Copenhagen, Denmark; 5 Bacteriology Department, Veterinary Sciences Division, Stormont, Belfast; 6 Veterinary Microbiology and Preventive Medicine, Iowa State University, Ames, Iowa, United States of America; 7 Chembio Diagnostic Systems, Medford, New York, United States of America; 8 UK Veterinary Laboratories Agency, Weybridge, New Haw, Addlestone, Surrey, United Kingdom; BMSI-A*STAR, Singapore

## Abstract

**Background:**

Early secretory antigenic target-6 (ESAT-6) and culture filtrate protein-10 (CFP-10) are co-secreted proteins of *Mycobacterium tuberculosis* complex mycobacteria (includes *M. bovis*, the zoonotic agent of bovine tuberculosis) involved in phagolysosome escape of the bacillus and, potentially, in the efficient induction of granulomas. Upon tuberculosis infection, multi-nucleate giant cells are elicited, likely as a response aimed at containing mycobacteria. In tissue culture models, signal regulatory protein (SIRP)α (also referred to as macrophage fusion receptor or CD172a) is essential for multi-nucleate giant cell formation.

**Methodology/Principal Findings:**

In the present study, ESAT-6/CFP-10 complex and SIRPα interactions were evaluated with samples obtained from calves experimentally infected with *M. bovis*. Peripheral blood CD172a^+^ (SIRPα-expressing) cells from *M. bovis*-infected calves proliferated upon in vitro stimulation with ESAT-6/CFP-10 (either as a fusion protein or a peptide cocktail), but not with cells from animals receiving *M. bovis* strains lacking ESAT-6/CFP-10 (i.e, *M. bovis* BCG or *M. bovis* ΔRD1). Sorted CD172a^+^ cells from these cultures had a dendritic cell/macrophage morphology, bound fluorescently-tagged rESAT-6:CFP-10, bound and phagocytosed live *M. bovis* BCG, and co-expressed CD11c, DEC-205, CD44, MHC II, CD80/86 (a subset also co-expressed CD11b or CD8α). Intradermal administration of rESAT-6:CFP-10 into tuberculous calves elicited a delayed type hypersensitive response consisting of CD11c^+^, CD172a^+^, and CD3^+^ cells, including CD172a-expressing multi-nucleated giant cells.

**Conclusions/Significance:**

These findings demonstrate the ability of ESAT-6/CFP-10 to specifically expand CD172a^+^ cells, bind to CD172a^+^ cells, and induce multi-nucleated giant cells expressing CD172a.

## Introduction

Tuberculosis (TB) in humans and animals may result from exposure to bacilli within the *Mycobacterium tuberculosis* complex (i.e., *M. tuberculosis, M. bovis, M. africanum, M. pinnipedi, M. microti, M. caprae*, or *M. canetti* [1]). *Mycobacterium bovis* is the species most often isolated from tuberculous cattle. Unlike *M. tuberculosis*, *M. bovis* has a wide host range, including several wildlife maintenance hosts for the infection in cattle. Early secretory antigenic target-6 (ESAT-6) and culture filtrate protein-10 (CFP-10) are co-secreted proteins of *M. tb* complex mycobacteria that form naturally a 1∶1 heterodimer upon export [2]. *esxA and esxB* genes encode ESAT-6 and CFP-10, respectively, and are located in the region of difference 1 (RD-1), an area of the virulent *M. tb* complex genome not present in the vaccine strain, *M. bovis* bacillus Calmette Guerin (BCG) and most other non-tuberculous mycobacteria [3], [4], [5], [6]. Widely utilized in diagnostic tests, ESAT-6 and CFP-10 are potent inducers of Th-1 cytokines [7]. ESAT-6 and CFP-10 are critical for TB pathogenesis [8] as removal of *esxA and esxB* genes from virulent *M. bovis* and *M. tuberculosis* results in attenuation and re-introduction of RD-1 to BCG *Pasteur* partially restores virulence [6], [9], [10], [11]. While ESAT-6 will disrupt lipid bilayers (indicating a cytolytic function [9], [12]), structural analysis of the ESAT-6/CFP-10 complex suggests another role more consistent with a receptor-mediated interaction with host cells [13]. Additionally, fluorescently tagged ESAT-6/CFP-10 binds human monocyte/macrophage tissue culture cells and this interaction is mediated by a long, flexible C-terminal arm on CFP-10 [13], [14]. With RAW cells, ESAT-6 interacts directly with TLR2 and inhibits signaling, thereby, dampening innate immune responses [15]. During *M. marinum* infection of zebrafish, macrophage aggregation is dependent upon RD-1 determinants [16], [17], further supporting a receptor-mediated interaction of ESAT-6/CFP-10 with host cells.

Signal regulatory protein (SIRP)α (also referred to as macrophage fusion receptor, CD172a or SHPS-1) is a transmembrane regulatory protein expressed primarily by myeloid cells (i.e., macrophages, monocytes, dendritic cells, granulocytes, myeloid progenitors), hematopoietic stem cells, and neurons [18], [19]. In the context of a potential role in TB pathogenesis, SIRPα is likely critical in the formation of multinucleate giant cells (as indicated by antibody blocking studies performed with in vitro models of giant cell formation [20], [21]) and in leukocyte trafficking via functional binding to the cell-associated ligand, CD47 [22], [23], [24]. Originally termed integrin-associated protein, CD47 is a broadly expressed member of the Ig superfamily (IgSF), essential for multiple key immune processes including phagocytosis, leukocyte migration, and self-recognition [25], [26], [27]. The extracellular region of SIRP family members (i.e., SIRPα, SIRPβ, and SIRPγ) consists of three joined IgSF domains, two IgC domains and a membrane-distal IgV domain [18], [28]. The IgV domain of SIRPα binds specifically to the single Ig-like domain on CD47, spanning a distance of ∼14 nm–typical of an immunological synapse [28]. The binding domain of SIRPα is analogous to hypervariable (CDR-like) regions of Ig and TCRs, presumably functioning as a sensitive recognition system for myeloid cell activation [28], [29]. One hypothesis is that SIRPs are closely related to germ-line rearranging antigen receptors, indicating a linkage between cell-mediated cytotoxicity and phagocytosis by cells expressing SIRPα. However, signaling via SIRPα is primarily inhibitory (the cytoplasmic portion of SIRPα contains four immunoreceptor tyrosine-based inhibititory motifs) to cell function, including phagocytosis [27], [30]. A scenario, in the context of TB, is that SIRPα-expressing cells phagocytose *Mycobacterium*-infected cells rendered apoptotic by specific T cell immunity. Upon apoptosis, CD47 expression is decreased on most cell types [31]; thereby, removing the ligand for the inhibitory SIRPα signal and permitting phagocytosis of the apoptotic cell by adjacent SIRPα-expressing cells [32]. Intriguingly, *M. tb* complex mycobacteria have multiple anti-apoptotic mechanisms, thereby, potentially subverting SIRPα/CD47-mediated killing mechanisms [reviewed in 33].

In the present study, ESAT-6/CFP-10 complex and SIRPα interactions were evaluated with samples obtained from calves experimentally infected with *M. bovis*. Our observations of in vitro stimulated peripheral blood mononuclear cells (PBMC) from *M. bovis*-infected cattle revealed consistent expansion of CD172a^+^ (SIRPα-expressing) cells upon stimulation with either a recombinant ESAT-6:CFP-10 fusion protein or a cocktail of ESAT-6 and CFP-10 peptides. Sorted CD172a^+^ cells from these cultures had a dendritic cell/macrophage morphology, bound fluorescently-tagged rESAT-6:CFP-10, bound and phagocytosed live *M. bovis* BCG, and co-expressed CD11c, DEC-205, CD44, MHC II, CD80/86, and a subset also co-expressed CD11b or CD8α. Intradermal administration of rESAT-6:CFP-10 into tuberculous calves elicited a delayed type hypersensitivity (DTH) response consisting of CD11c^+^, CD172a^+^, and CD3^+^ mononuclear cell infiltrates, including CD172a-expressing multi-nucleated giant cells. These novel findings demonstrate the ability of ESAT-6/CFP-10 to specifically expand CD172a^+^ cells, bind to CD172a^+^ cells, and induce multi-nucleated giant cells expressing CD172a.

## Materials and Methods

### Animals, vaccination, and challenge procedures

Twenty nine male Holstein calves of approximately 3 months of age were obtained from a TB-free herd in Iowa or Wisconsin, USA and housed at the National Animal Disease Center in Ames, Iowa according to institutional guidelines, approved animal care and use protocols and the National Institutes of Health guide for the care and use of laboratory animals. Approval of animal protocols was by the USDA, NADC animal care and use committee. Treatment groups included: non-infected/non-vaccinated controls (n = 3), virulent *M. bovis*-infected (10^5^ cfu by aerosol, n = 6; 10^3^ cfu by aerosol, n = 14), *M. bovis* BCG-vaccinated (Pasteur strain, n = 3), and ΔRD1 *M. bovis*-vaccinated (Ravenel background, n = 3) calves. The ΔRD1 *M. bovis* vaccine [34] was prepared by targeted mutagenesis as described [9]. Vaccines (BCG and ΔRD1 *M. bovis*) were administered subcutaneously at 2 wks of age. Virulent *M. bovis* for challenge [95–1315, USDA, Animal Plant and Health Inspection Service (APHIS) designation] was originally isolated from a white-tailed deer in Michigan, USA [35]. The challenge inoculum was administered at either 2.5 months (10^3^ cfu group, n = 14) or 6 months (10^5^ cfu group, n = 6) of age by aerosol as described [36].

### Cell culture, dye tracking, cell sorting, and flow cytometry

Peripheral blood mononuclear cells (PBMC) were isolated by density gradient centrifugation of peripheral blood buffy coat fractions collected into 2× acid citrate dextrose. Staining of PBMC with PKH67 was performed according to manufacturer instructions (Sigma, St. Louis, Missouri) and as described [37]. Briefly, 2×10^7^ PBMC were centrifuged (10 min, 400×*g*), supernatants aspirated, and cells resuspended in 1 ml of diluent provided in the PKH67 kit. Cells in diluent were added to 1 ml of PKH67 green fluorescent dye (2 µM) and incubated 5 min followed by a 1 min incubation with 2 ml of fetal bovine sera (FBS, National Veterinary Services Laboratory, Ames, Iowa) to adsorb the excess dye and stop further dye uptake by cells. Individual wells of 96-well round-bottom microtiter plates (Falcon, Becton-Dickinson; Lincoln Park, New Jersey) were then seeded with 5×10^5^ PBMC in a total volume of 200 µl per well. Medium was RPMI 1640 (GIBCO, Grand Island, New York) supplemented with 2 mM L-glutamine, 25 mM HEPES buffer, 100 units/ml penicillin, 0.1 mg/ml streptomycin, 1% non-essential amino acids (Sigma), 2% essential amino acids (Sigma), 1% sodium pyruvate (Sigma), 50 mM 2-mercaptoethanol (Sigma), and 10% (v/v) FBS. In vitro treatments included medium plus 1 µg/ml recombinant Mobility Protein of Bovis (rMPB)-83 (MPB83 is an immunodominant antigen of *M. bovis* used as a recombinant antigen control), 1 µg/ml ESAT-6 and CFP-10 peptides, 1 µg/ml rESAT-6:CFP-10 [38], or medium alone (no stimulation). Cultures were incubated for 6 d at 39°C and 5% CO_2_ in air.

Phenotype analysis of PBMC was performed as described previously [37]. Briefly, cells were harvested and incubated with 1 µg primary monoclonal antibodies/10^6^ cells (mAb's; DH59B, CD172a; CACT116A, CD25; CACT114A, CD26; BAT31A, CD44; BAQ92A, CD62L; MM1A, CD3; GC50A1, CD4; BAQ111A, CD8α; BAT82A, CD8β; CACT61A and GB21A, γδ TCR; PIG45A, IgM (B cell); BAQ44A, BB2 (B cell); BAQ153a, CD11c; CAM36A, CD14; TH14B, MHC class II; CD11b, MM12A obtained from VMRD, Pullman Washington or CC149, CD172a; CC8, CD4; MCA1651, DEC-205; MCA2365, CD335 (NK cell) obtained from Serotec, Kidlington, UK at room temperature for 15 min. Cells were then washed and stained with isotype appropriate goat anti-mouse phycoeythrin- (PE, Southern Biotechnology Associates, Birmingham, Alabama), or Peridinin Chlorophyll Protein- (PerCP, Becton Dickinson) conjugated secondary antibodies or hCTLA4/Ig-PE for CD80/86 (Ancel, Bayport, Minnesota) at room temperature for 15 min. For studies not using PKH67, isotype appropriate goat anti-mouse fluorescein isothiocyanate- (FITC, Southern Biotechnology Associates) conjugated secondary antibodies were also used. Three-color flow cytometric analysis was performed using a FACScan (BD Biosciences, San Jose, California, at least 25,000 events within the live cell gate captured per sample) flow cytometer. Data were analyzed with either Cellquest Pro (BD Biosciences) or FlowJo (Tree Star Inc., San Carlos, California) software.

For cell sorting, cells from 6 day rESAT-6:CFP-10-stimulated cultures (n = 2, 3 months after *M. bovis* infection) were harvested from treatment replicates in 96-well tissue culture plates (2×10^8^ cells/animal) and labeled on ice with 32 µg DH59B primary antibody and 128 µg goat anti-mouse IgG1-PE for sorting of CD172a^+^ cells using a FACS-Aria (Becton Dickinson, >98% purity). Sorted CD172a^+^ cells were evaluated by transmission electron microscopy and fluorescence microscopy.

### Electron microscopy

Sorted 172a+ cells were fixed by suspension in 2.5% glutaraldehyde in 0.1 M cacodylate buffer at 4°C. After 2 hours fixation, cells were rinsed in cacodylate buffer, postfixed in 1% osmium tetroxide, dehydrated in alcohols, cleared in propylene oxide, and embedded in epoxy resin. Ultrathin sections of appropriate areas were cut, stained with uranyl acetate and lead citrate, and examined with a FEI Tecnai 12 Biotwin (FEI company, Hillsboro, OR) transmission electron microscope.

### Fluorescence Microscopy

Sorted CD172a^+^ cells were re-suspended in supplemented RPMI and incubated in 96 well plates (10^6^ cells/well) with either *M. bovis* BCG-FITC (cell : mycobacteria ratio at 1∶1 and 1∶3) or rESAT-6:CFP-10-FITC (cell : µg protein ratio at 1∶1 and 1∶10) for 2, 24, and 48 hr. Fluorescein-conjugation was via a Fluorotag FITC Conjugation kit (Sigma). After the respective incubation period, non-bound bacteria/protein was removed by two washes in PBS. Washed cells were transferred to cytospin slides (Thermo Fisher Scientific, Waltham, Massachusetts), centrifuged, and coverslips mounted using Prolong Gold, anti-fade reagent (Molecular Probes, Eugene, Oregon). Fluorescent images were analyzed with a light microscope (Nikon Eclipse E800; Nikon Co., Tokyo, Japan) equipped with the VFM Epi-fluorescent attachment with xenon lamp (Nikon, Co.). Images were captured using a microscope mounted digital camera (Spot RT, Diagnostic Instruments Inc., Sterling Heights, MI, USA).

### Immunohistochemistry

Samples were collected from intradermal injection sites and snap frozen in liquid nitrogen cooled isopentane and stored at −80°C. Frozen sections were cut by cryostat in 6 µm sections and processed for immunohistochemistry using primary antibodies to the cell markers CD4, ILA11; CD8, BAQ11A; γ/δ T cell receptor, GB21A; CD3, MM1A; CD172a, DH59B; and CD11c as described previously [39], [40] using HistoMark Biotin Streptavidin-HRP system (Kirkegaard and Perry, Gaithersburg, MD, USA) and 3,3′ diaminobenzidine-nickel (DAB-Ni peroxidase substrate, Vector Laboratories, Burlingame, CA, USA) as a peroxidase substrate. Non-specific protein binding was blocked using normal goat serum and endogenous peroxidase activity was quenched using 0.3% H_2_O_2_ in methanol prior to application of the primary antibody. Digital images of sections from all palatine tonsils were obtained with a light microscope and digital camera.

#### Statistics

Data were analyzed by one-way analysis of variance followed by Tukey-Kramer multiple comparisons test using a commercially available statistics program (InStat 2.00, GraphPAD Software, San Diego, Calif.).

## Results

### In vitro expansion of CD172a^+^ cells in response to ESAT-6/CFP-10 stimulation

**Figure 1:**
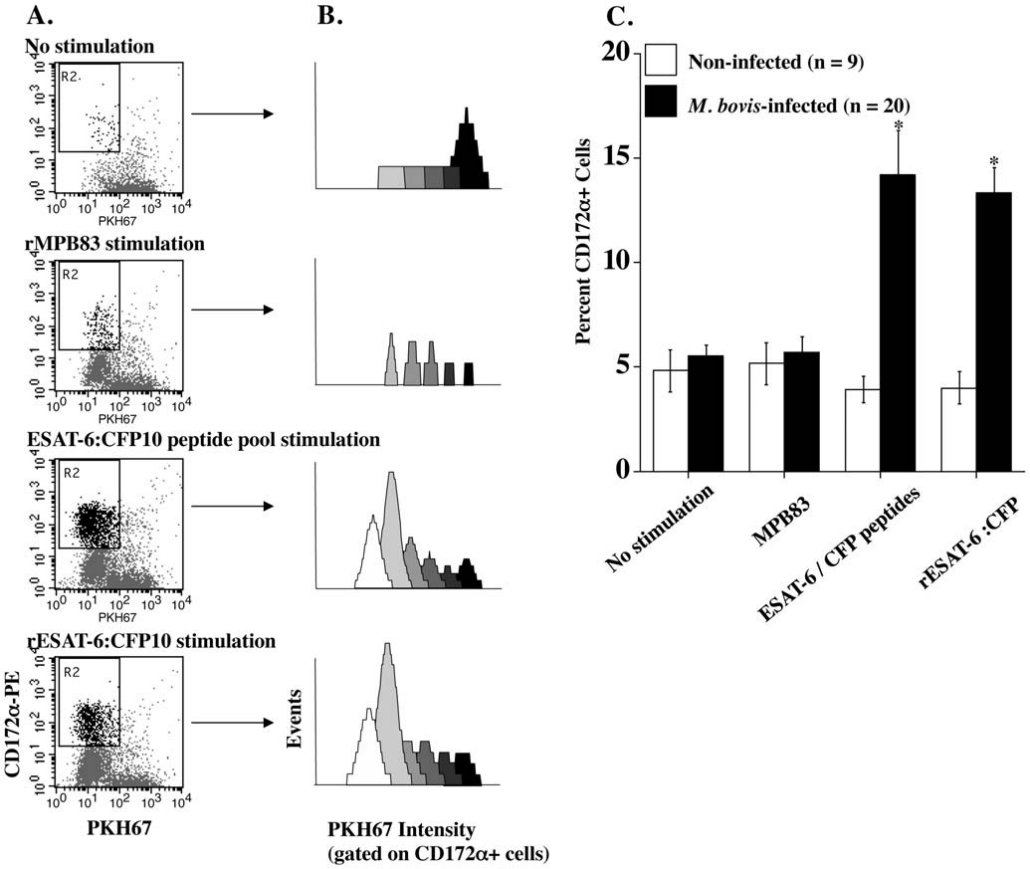
Expansion of CD172a^+^ cells in response to ESAT-6/CFP-10 stimulation. Isolated PBMC were stained with PKH67 and cultured for 6d with media only, rMPB83, a pool of overlapping ESAT-6 and CFP-10 peptides, or rESAT-6:CFP-10. Data are depicted as: (A) dot plots (CD172a-PE (y-axis) versus PKH67 (x-axis, green fluorescence), (B) histograms generated by Modfit Proliferation Wizard analysis of PKH67 staining intensity (gated on CD172a-PE^+^ cells within the live gate) and (C) mean (±SEM) percent CD172a^+^ cells within PBMC cultures (stimulation indicated in the lower margin) from non-infected (open bars, n = 9, includes non- (n = 3), BCG- (n = 3), and ΔRD1- (n = 3) vaccinates) or *M. bovis*-infected (closed bars, n = 20) cattle. Responses did not differ between controls, BCG- and ΔRD1- vaccinates; thus, these groups were combined. Gate R2 in panel A highlights the CD172a^+^, PKH67^lo^ proliferative fraction. For panels A and B, data from a single *M. bovis*-infected animal are provided that are indicative of a representative response.

In vitro stimulation of peripheral blood leukocytes from TB patients with ESAT-6 and/or CFP-10 peptides or recombinant protein(s) elicits a specific T cell proliferative and cytokine response utilized extensively for TB diagnosis [41]. Extending these observations, present findings demonstrate in vitro expansion of CD3^−^, CD172a^+^ cells in response to ESAT-6/CFP-10 stimulation of PBMC from tuberculous cattle (Table 1). Stimulation of PKH67-labeled PBMC with ESAT-6/CFP-10 resulted in an increase in CD172a^+^/PKH67^lo^ cells as compared to non-stimulated cultures, indicating generation of CD172a^+^ daughter (proliferative) fractions (Fig. 1A and B). With TB-infected cattle, percentages of CD172a^+^ cells in cultures stimulated with either the recombinant fusion protein or an ESAT-6/CFP-10 peptide cocktail (∼14%, Fig. 1C) exceeded (P<0.05) CD172a+ percentages in cell populations from non-infected, BCG-vaccinated, and ΔRD-1-vaccinated cattle (∼4%, Fig. 1C). Both BCG and ΔRD-1 attenuated *M. bovis* vaccine strains lack ESAT-6, CFP-10, and select ESX-1 secretion apparatus genes; thus, these strains do not produce ESAT-6 or CFP-10. Stimulation with another immunodominant antigen of *M. bovis* (i.e., MPB83) did not result in expansion of CD172a^+^ cells (Table 1, Fig. 1), despite significant proliferation of other cell types (CD172a^−^/PKH67^lo^ in Fig. 1A and data not shown) to MPB83 stimulation. These findings demonstrate that ESAT-6/CFP-10 stimulation of PBMC from TB-infected cattle results in an environment conducive to the proliferation and/or maturation of CD172a^+^ cells.

**Table 1:** In vitro expansion of CD3^−^, CD172a^+^ cells within PBMC cultures from *M. bovis* infected cattle stimulated with ESAT-6/CFP-10.

Group	Media only	rMPB83	ESAT-6/CFP10 peptides	rESAT-6:CFP10
**Non-Infected (n = 3)**	3.1 (1.4)	4.1 (1.7)	1.4 (0.4)	2.2 (1.0)
**BCG-vaccinated (n = 3)**	3.0 (1.0)	3.0 (0.6)	2.2 (0.4)	2.6 (0.7)
**ΔRD1-vaccinated (n = 3)**	2.6 (1.1)	4.7 (1.5)	2.1 (0.5)	1.9 (0.8)
***M. bovis*** **-infected (n = 3)**	2.5 (0.2)	3.8 (0.6)	11.7 (1.7)**	10.5 (0.9)**

a Data are presented as mean (±standard error) percent of CD3^−^, CD172a^+^, PKH67^lo^ cells (R2 gate in Fig. 1A). Isolated mononuclear cells were stained with PKH67 (a green fluorescent dye used for cell proliferation analysis), cultured for 6 days with or without stimulation as indicated in the upper margin, and analyzed by flow cytometry for phenotype and PKH67 staining intensity. Both BCG and ΔRD-1 attenuated *M. bovis* vaccine strains lack ESAT-6, CFP-10, and select ESX-1 secretion apparatus genes; thus, these strains do not produce ESAT-6 or CFP-10. In contrast to ESAT-6/CFP10, stimulation with MPB83, another immunodominant antigen of *M. bovis*, does not result in expansion of CD172a^+^ cells.

### Phenotype of CD172a^+^ cells responsive to ESAT-6/CFP-10 stimulation

**Figure 2:**
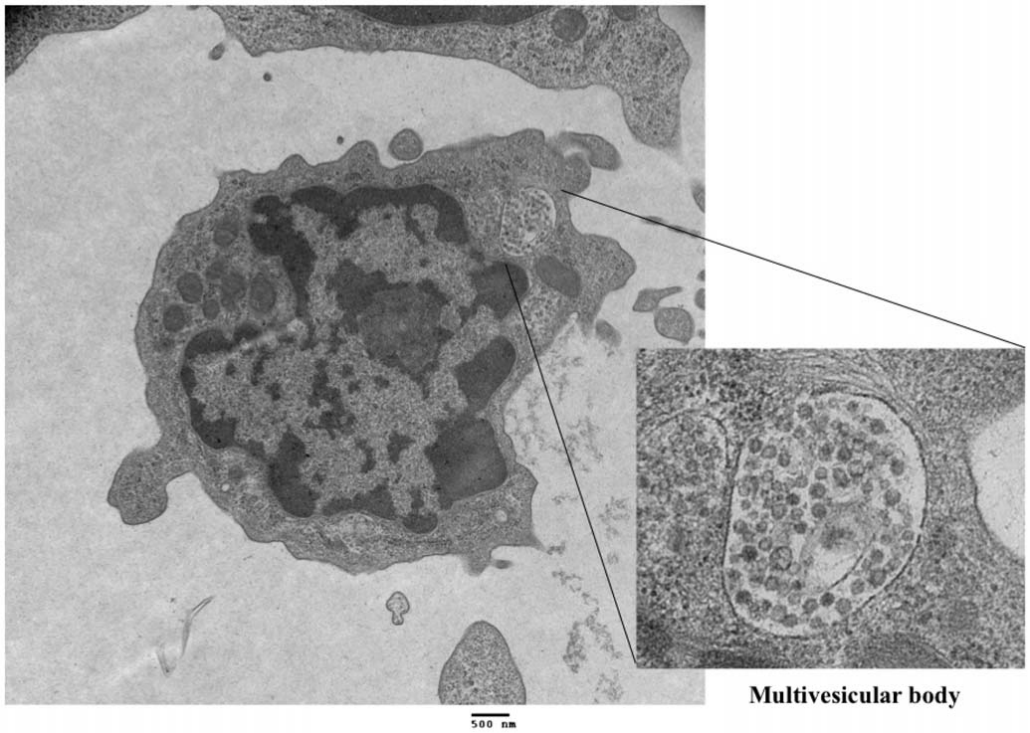
Ultrastructure of CD172a cells. CD172a^+^ cells were isolated by high-speed cell sorting (>98% purity) from PBMC cultures stimulated with rESAT-6:CFP-10 for 6d and evaluated by transmission electron microscopy. A cell with morphology representative of the majority of the CD172a^+^ cells within the culture is depicted. Bar in lower margin indicates scale. The higher magnification inset is a multi-vesicular body.

Various dendritic cell (DC) populations are defined for cattle [42], [43], [44], [45], including a subset of myeloid DC's expressing SIRPα (i.e., CD172a^+^) that bind CD47 [46]. By flow cytometry, CD172a^+^ cell populations responding to ESAT-6/CFP-10 stimulation in vitro did not express bovine CD3, CD4, CD8β, CD14, CD25, CD26, CD62L, γδ TCR, or CD335 (NK cells); however, they did express CD11c, CD44, CD80/86(^lo^), MHC class II (^lo^), DEC-205 with a subset expressing CD11b or CD8α. To define the morphology of ESAT-6/CFP-10 expanded CD172a^+^ cells, an enriched fraction of CD172a^+^ cells (>98% purity) were obtained via high-speed cell sorting of rESAT-6:CFP-10 stimulated cultures (6d) and evaluated by transmission electron microscopy (Fig. 2). Characteristics of sorted CD172a^+^ cells included: 8–16 µm in diameter, irregular shaped cell margin, multiple mitochondria, 0.5–2.5 µm long cytoplasmic processes, high nucleus to cytoplasm ratio, irregular contoured nucleus, prominent nucleolus, and presence of myelin figures and multivesicular bodies (inset in Fig. 2). These characteristics are consistent with a myeloid cell lineage.

### Binding of rESAT-6:CFP-10 and M. bovis BCG to CD172a^+^ cells

**Figure 3:**
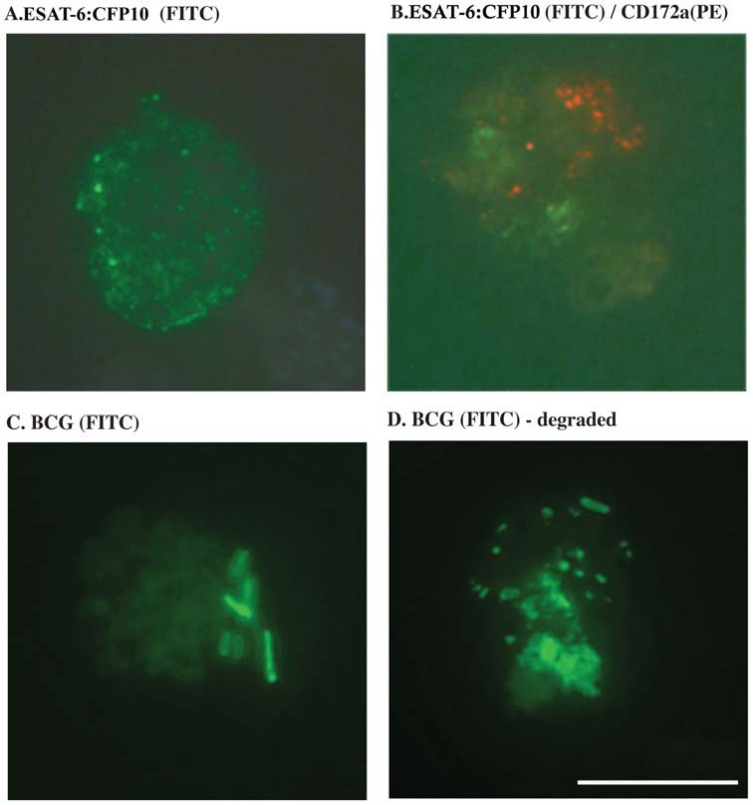
CD172a cells bind rESAT-6:CFP-10 and *M. bovis* BCG. CD172a^+^ cells (PE-labeled) were isolated by high-speed cell sorting (>98% purity) from rESAT-6:CFP-10-stimulated PBMC 6d cultures; incubated with rESAT-6:CFP-10-FITC or BCG-FITC for 2–96 hrs; and evaluated by fluorescence microscopy. (A) rESAT-6:CFP-10-FITC bound to the surface of CD172a^+^ cells in a focal pattern (24 hr cultures shown). Labeling patterns were similar at 2, 24 (shown in Fig. 3A) and 96 hrs after addition of rESAT-6:CFP-10 (FITC) to cells, except for increased polarization of staining at 96 hrs. (B) Over the 96 hr culture period, PE-staining (red) used for CD172a^+^ cell sorting persisted (24 hr cultures shown) with a polar distribution, possibly due to capping of antibody bound to CD172a. However, rESAT-6:CFP-10-labeling (green) did not overlap with CD172a-PE labeling (red). (C) *M. bovis* BCG was detected in association with CD172a^+^ cells at 2 (not shown) and 24 hrs (green, intact bacteria associated with the cell surface, panel C) after addition of the live bacteria to the CD172a^+^ cell culture and by 96 hrs, *M. bovis* BCG was internalized and mostly degraded (D). White bar = 10 µm.

A proposed function of the ESAT-6/CFP-10 complex is that it binds mononuclear cells and acts as a signaling molecule [13]; however, specific cell types that the complex binds to (especially in the context of TB infection) are not known. To evaluate the potential for direct interaction of CD172a^+^ cells with the fusion protein, expanded CD172a^+^ cells were sorted from rESAT-6:CFP-10-stimulated (6d) PBMC cultures, incubated with rESAT-6:CFP-10-FITC for 2–96 hrs, and evaluated by fluorescence microscopy (Fig. 3). Within 2 hrs, the fluorescently-tagged protein bound to the surface of CD172a^+^ cells in a focal pattern (Fig. 3A); however, rESAT-6:CFP-10-FITC labeling did not overlap with CD172a-PE labeling (Fig. 3B), indicating that the fusion protein does not likely interact with CD172a directly. Labeling patterns were similar at 2, 24 and 96 hrs after addition of rESAT-6:CFP-10 (FITC) to cells, except for increased polarization of staining at 96 hrs. Over the 96 hr culture period, PE-staining used for CD172a^+^ cell sorting persisted. CD172a-labeling was polar in distribution, possibly due to capping of antibody bound to CD172a. Sorted CD172a^+^ cells also bound (Fig. 3C, 24 hrs after culture), internalized, and degraded *M. bovis* BCG (Fig. 3D, 96 hrs after culture); indicating their potential for in vivo phagocytosis of mycobacteria. Together, these findings identify a role for CD172a^+^ cells in the response to bovine TB and elucidate a cell type to which rESAT-6:CFP-10 binds.

### In vivo response to rESAT-6:CFP-10

**Figure 4:**
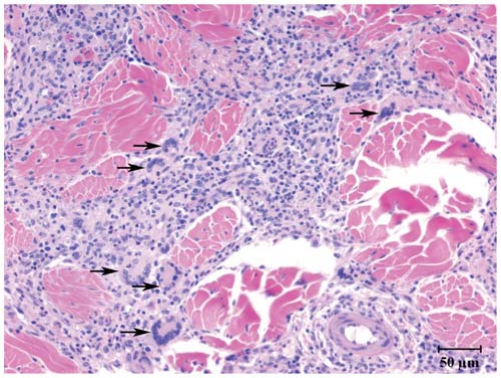
rESAT-6:CFP-10 induces granulomatous inflammation with multi-nucleated giant cells. *Mycobacterium bovis*-infected cattle (n = 5) received 400 µg rESAT-6:CFP-10 intradermally and reactions were characterized after 6 days. Injection sites were collected at necropsy, fixed in formalin, and stained with hematoxylin and eosin. Injection sites consisted of predominately mononuclear cell infiltrates with intermittent, yet consistently detected, multi-nucleated giant cells (arrows). Prior studies have demonstrated that the inflammatory response to rESAT-6 is specific to *M. bovis* infection [48]. Also, intradermal injection of a BCG-vaccinated calf with 400 µg rESAT-6:CFP-10 did not elicit a DTH response and multi-nucleated giant cells were not detected within the injection site biopsy from this negative control animal.

**Figure 5:**
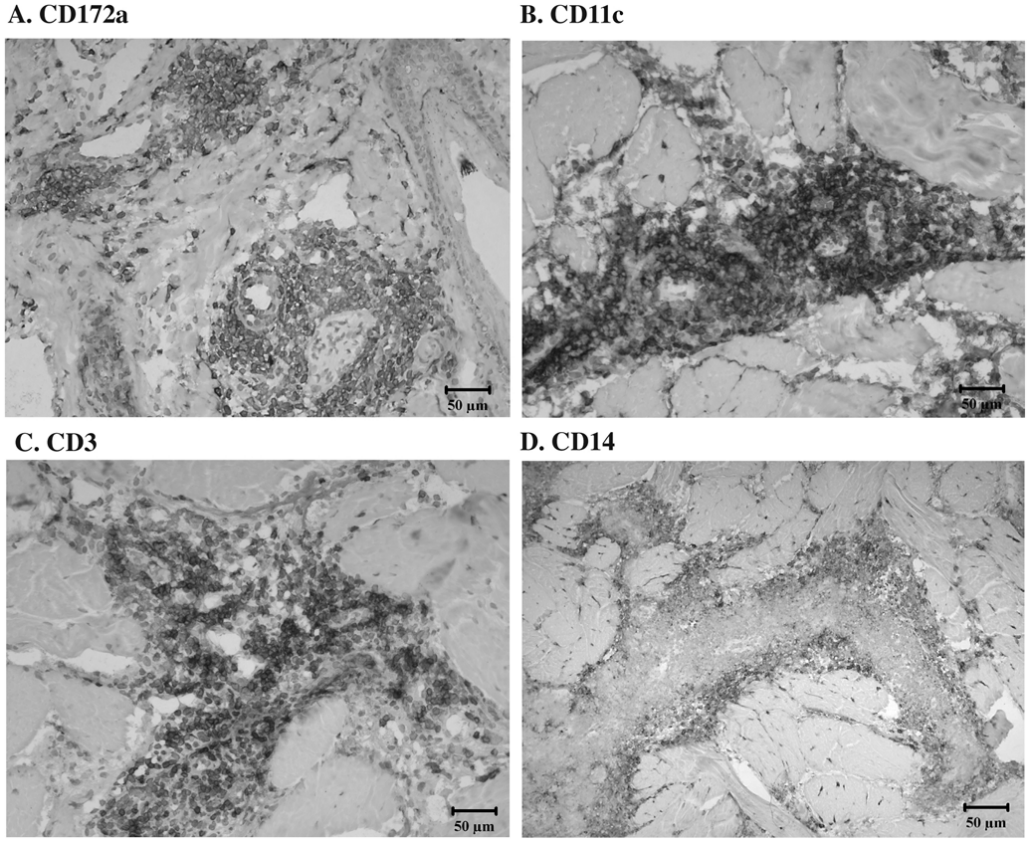
CD172a cells infiltrate rESAT-6:CFP-10 injection sites. *Mycobacterium bovis*-infected cattle (n = 5) received 400 µg rESAT-6:CFP-10 intradermally and reactions were characterized after 6 days. Injection sites were collected at necropsy, snap frozen, and evaluated by immunohistochemistry for expression of (A) CD172a, (B) CD11c, (C) CD3, and (D) CD14 on cellular surfaces. Few B cells or γδ T cells were detected (data not shown).

**Figure 6:**
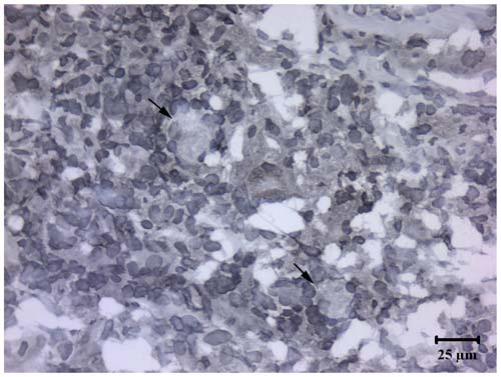
Multinucleate giant cells elicited by rESAT-6:CFP10 are composed of a ring of CD172a^+^ cells. *Mycobacterium bovis*-infected cattle (n = 5) received 400 µg rESAT-6:CFP-10 intradermally and reactions were characterized after 6 days. Injection sites were collected at necropsy, snap frozen, and evaluated by immunohistochemistry for expression of CD172a. Arrows depict a CD172a^+^ multi-nucleate giant cell consisting of a ring of peripherally located nuclei and abundant cytoplasm located centrally.

Originally termed macrophage fusion receptor, CD172a was the first protein identified as essential for macrophage fusion in tissue cultures [reviewed in 47]. To extend in vitro findings on ESAT-6/CFP-10 and CD172a interactions, *M. bovis*-infected cattle (n = 5) were injected with 400 µg rESAT-6:CFP-10 intradermally and reactions characterized. Prior studies have demonstrated that this response in cattle is specific to *M. bovis* infection [48]. Indeed, intradermal injection of rESAT-6:CFP-10 to a BCG-vaccinated calf in the present study did not elicit a DTH response and multi-nucleated giant cells were not detected within a biopsy of the injection site. With *M. bovis*-infected cattle (n = 5), rESAT-6:CFP-10 elicited a DTH response characterized by infiltrates consisting of predominately mononuclear cells with intermittent, yet consistently detected, multi-nucleated giant cells (Fig. 4). Infiltrates consisted primarily of CD3^+^, CD14^+^, CD11c^+^, and CD172a^+^ cells (Fig. 5). In general, lymphocyte infiltrates were primarily CD4^+^ cells with lesser numbers of CD8^+^ cells and few B or γδ T cells. Spatiotemporally, CD11c^+^, CD172a^+^ and CD3+ cells were all located in dense perivascular accumulations that extended outward separating and dividing collagen bundles and adnexal structures. In contrast, CD14^+^ cells were located at the periphery of mononuclear cell infiltrates (Fig. 5D). Of particular note, multi-nucleated giant cells were composed of a concentric ring of CD172a^+^ expression, albeit, the central cytoplasmic core of each of the giant cells was devoid of CD172 staining (Fig. 6). Subcutaneous inoculation of naïve cattle with either virulent *M. bovis*, *M. bovis* BCG, or ΔRD1 *M. bovis* (n = 1/group) also resulted in granulomatous reactions containing multi-nucleated giant cells, likely due to the presence of envelope glycolipids in each of these live inocula [49]. Thus, ESAT-6/CFP-10 is sufficient for the induction of multi-nucleated giant cells in TB-infected animals but is not required, as attenuated live mutants lacking ESAT-6/CFP-10 also elicited multi-nucleated giant cells.

## Discussion

Multiple functions are proposed for ESAT-6 and CFP-10 proteins produced by *M. tb*-complex mycobacteria [reviewed in 16]. ESAT-6 interacts with biomembranes after dissociation from its putative CFP-10 chaperone within the acidic phagolysosome [12]; thereby affording a “phagolysosome escape” mechanism for the pathogen. However, dissociation of the ESAT-6/CFP-10 complex under acidic conditions is unclear as a recent study indicated that the complex is stable at a pH of 4.5 [50] while another study demonstrated that the complex dissociates between a pH of 4 and 5 [12]. Regardless, ESAT-6 deletion mutants of *M. tb* have reduced tissues invasiveness, likely due to a loss of cytolytic activity [9]. With the *M. marinum*/zebrafish granuloma model, RD1 components are also required for efficient recruitment of macrophages to granulomas for phagocytosis of dead macrophages with viable mycobacteria [51], thus, “creating new bacterial growth niches” [17]. RD-1 proteins, including ESAT-6/CFP-10, likely elicit a faster kinetics of granuloma formation offering a distinct growth advantage for the pathogen [51]. In addition to enhancing recruitment of cells susceptible to infection, the stable ESAT-6/CFP-10 complex binds to host cells [13]; subsequently, modulating the host response favorably for the pathogen potentially via down-regulation of host cell killing mechanisms and immune cell activation [52]. While a specific receptor for ESAT-6 has been identified using monocyte/macrophage cell lines [15], specific cell types to which ESAT-6/CFP-10 binds within a host have not previously been determined, particularly in the context of TB infection. Present findings support a specific interaction of the ESAT-6/CFP-10 complex with bovine CD172a-expressing cells. Stimulation of PBMC cultures from *M. bovis*-infected calves with ESAT-6/CFP-10 resulted in the specific expansion of CD172a^+^ cells and the fusion protein bound to the surface of CD172a^+^ cells. Further studies are necessary to characterize molecular interactions of ESAT-6/CFP-10 with CD172a^+^ cells and the ramifications of this protein/cell interaction.

SIRPα-CD47 interactions are essential for efficient migration of DC's to skin [53] and secondary lymphoid organs [54]. Thus, ESAT-6/CFP-10-induced expansion of CD172a (SIRPα)-expressing cells may favor migration of DC/macrophage trafficking to infection sites; thereby, promoting efficient granuloma formation and early dissemination of *M. tb* complex mycobacteria, as proposed for RD1 components by Davis and and Ramakrishnan [51]. Current findings demonstrate that injection of rESAT-6:CFP-10 elicited granulomatous inflammation with infiltration of numerous T cells, CD172a^+^ and CD14^+^ cells in *M. bovis*-infected calves; further supporting a role for ESAT-6/CFP-10 in the recruitment of naïve cells for infection and granuloma formation. A unique aspect of the cellular infiltrates of rESAT-6:CFP-10 injection sites was the presence of numerous multi-nucleated giant cells. These cells provide an opportunity for the host to resorb large substances (e.g., bacteria) with an enhanced capacity (as compared to mononucleate cells) via an extracellular lysosome mechanism [23], [55]. Multi-nucleate giant cell formation is mediated, in part, by macrophage fusion receptor, also termed CD172a or SIRPα. Cell surface expression of CD172a is strongly and transiently induced upon giant cell formation. As opposed to phagocytosis, SIRPα-CD47 interactions provide “self recognition” signals that prevent killing of internalized (i.e., fused) cells. As with mouse and human cell lines [reviewed in 23], present findings demonstrate that bovine multi-nucleate giant cells also express CD172a. Additionally, ESAT-6/CFP-10 was sufficient for induction of giant cells in TB-infected calves. Numerous other components of the tubercle bacillus may also induce giant cell formation [49]; however, this is the first observation that a defined protein antigen, ESAT-6/CFP-10, induces these cells without support from mycobacterial glyco- or phospho-lipids, potentially via a CD172a-mediated mechanism. Based on these findings and recently published observations, an important yet complex question arises: Does induction of multi-nucleate giant cells by ESAT-6/CFP-10 benefit the pathogen, host, or is it a compromise of this intricate interaction?

In the present study, ESAT-6/CFP-10 stimulation of PBMC from TB-infected cattle resulted in an environment conducive to the proliferation and/or maturation of CD172a^+^ cells. In vitro expansion of CD172a^+^ cells most likely resulted from indirect stimulation via growth factors/cytokines produced by ESAT-6/CFP-10-specific T cells. Indeed, rESAT-6:CFP-10 elicits robust *M. bovis*-specific CD4 and CD8 proliferative responses associated with increased expression of activation markers including CD25, CD26, and CD45RO by responding T cells [57]. With this response, antigen-presenting cells required to support specific T cell responses may have included CD172a^+^ cells. Another possibility is that direct interaction of ESAT-6/CFP-10 with CD172a^+^ cells elicited the response. In addition to IFN-γ, rESAT-6:CFP-10 stimulation of PBMC from TB-infected cattle elicits potent TNF-α and IL-4 responses [56], each supportive of myeloid cell maturation. Additionally, TNF-α produces pleiotropic effects in relation to TB granuloma formation and mycobacterial control [reviewed and modeled in 58]. Further, IL-4 induces multi-nucleated giant cell formation in vitro [59]. Likewise, rESAT-6:CFP-10-specific MIP-1α production by bovine mononuclear cells [56] may contribute to trafficking of CD172a^+^ cells into rESAT-6:CFP-10 injection sites. With infection, continued secretion of ESAT-6/CFP-10 by *M. tb* complex mycobacteria would lead to T cell production of cytokines/chemokines/growth factors that support trafficking and expansion of CD172a^+^ cells within lesions. Further studies, however, are warranted to determine specific biologic messengers facilitating these responses.

Resolution of the solution structure for the ESAT-6/CFP-10 complex to high precision has provided clear evidence for a long flexible C-terminal arm on CFP-10 necessary for binding to monocyte lineage human cell lines [13]. Present findings build upon this observation by demonstrating that the ESAT-6/CFP-10 complex binds to CD172a^+^ cells and is sufficient for the induction of multi-nucleated giant cells in TB-infected animals.
